# The Potential Role of Auditory Nerve Development in Sensorineural Hearing Loss: from Pathophysiology to Treatment Insights

**DOI:** 10.7150/ijms.131458

**Published:** 2026-03-25

**Authors:** Li Tian, Juanjuan Li, Yun Hu, Mengdi Li, Wenwen Xie, Jiaxue Feng, Peng Zhang, Xianhai Zeng

**Affiliations:** 1 Department of Graduate and Scientific Research, Zunyi Medical University Zhuhai Campus, Zhuhai, Guangdong, China.; 2 Department of Otolaryngology, Shenzhen Longgang Otolaryngology hospital & Shenzhen Otolaryngology Research Institute, Shenzhen, Guangdong, China.

**Keywords:** sensorineural hearing loss, hearing disorders, auditory nerve, regulatory mechanisms, treatment strategies

## Abstract

Sensorineural hearing loss (SNHL), as one of the most common types of hearing impairment, has seen a continuous increase in global incidence. It has seriously affected patients' quality of life and is closely associated with a range of psychological and mental health issues, while also posing a significant burden on healthcare systems. It is known that the etiologies of SNHL mainly include environmental factors (such as noise exposure, ototoxic drugs, and viral infections), genetic factors (including relevant gene mutations), and age-related degenerative changes in the auditory system (presbycusis). These pathogenic factors, whether acting individually or synergistically, can interfere with the normal development and function of the auditory nerves, resulting in damage to cochlear hair cells and auditory neurons, thereby causing irreversible hearing impairment. Therefore, a deep understanding of the regulatory mechanisms of auditory nerve development, structural and functional maturation, as well as its survival capacity and plasticity changes after injury, is crucial for elucidating the pathological basis of SNHL heterogeneity and promoting the development of precise treatment strategies. This article systematically reviews the molecular basis of auditory nerve development, the related pathological mechanisms, and causes of injury, while also exploring the cutting-edge therapeutic advances in this field, aiming to provide new insights for clinical interventions in sensorineural hearing loss and ultimately improve patients' auditory function and quality of life.

## 1. Introduction

Hearing loss has become an increasingly severe public health challenge worldwide. According to the World Health Organization's latest World Hearing Report, it is estimated that by 2050, nearly 2.5 billion people globally will experience some degree of hearing impairment, with approximately 700 million requiring rehabilitation and medical services such as otology and hearing care^1^, ^2^. As a common sensory disorder, hearing loss not only severely affects patients' ability to communicate verbally, but is also closely associated with negative health outcomes such as depression, social isolation, and cognitive decline, significantly reducing quality of life and imposing a heavy socioeconomic burden^3^. Hearing loss can be classified as sensorineural, conductive, or mixed, depending on whether the damage affects sound conduction or perception^4^. SNHL is the most common type of hearing impairment, and its pathological basis stems from irreversible damage to cochlear sensory hair cells, the stria vascularis, spiral ganglion neurons (SGNs), and/or synaptic connections in the auditory pathway^3^, ^5^, ^6^. Normal conduction of auditory signals relies on a multi-level conversion process from the peripheral to the central system: sound is transmitted to the cochlea through the outer and middle ears, where sensory hair cells in the cochlea convert mechanical vibrations of the basilar membrane into electrical neural signals. These signals are then transmitted via SGNs to the cochlear nucleus in the brainstem, and ultimately ascend to the auditory cortex for higher-level processing^7^. In this precisely regulated pathway, SGNs serve as a crucial hub connecting peripheral receptors with the central system, and the integrity of their structure and function is essential for the accurate transmission of sound signals. Recent studies have shown that noise exposure, ototoxic drugs, and viral infections, gene mutations, as well as the aging process can all lead to abnormal development or dysfunction of the auditory nervous system, thereby triggering SNHL^8^. It is noteworthy that mammalian cochlear hair cells and SGNs lack regenerative ability, a characteristic that makes SNHL a permanent damage once it occurs^3^, ^9^.

Currently, the main clinical interventions for SNHL include ear reconstruction, hearing aids, and conventional cochlear implantation. However, these treatments can only improve patients' hearing to a certain extent by amplifying sound or using electrical stimulation to replace the function of damaged hair cells, and they cannot fundamentally repair the damaged auditory neural network. Therefore, there are significant limitations in their clinical application^10^. As the main hub of the peripheral and central auditory system, the number and synaptic integrity of SGNs directly determine the transmission of sound signals. In common causes of SNHL, degeneration of SGNs is often an irreversible step, making the extent of their damage a better predictor of final hearing outcomes than hair cells. Therefore, deeply elucidating the molecular regulatory mechanisms of auditory nerve development, systematically analyzing the target sites and pathological mechanisms of damaging factors, and exploring innovative therapeutic strategies based on this knowledge may provide new theoretical foundations and technical approaches for the precise treatment of SNHL, ultimately improving patients' hearing rehabilitation outcomes and quality of life.

## 2. The Development and Maturation of the Auditory Nerve

### 2.1 Embryonic origin and ganglion formation

All sensory organs of the inner ear and their associated sensory ganglia originate from the same embryonic structure-the otic placode^11^. After the three germ layers are established at the gastrula stage, the Bone Morphogenetic Protein signaling gradient determines the transformation of the ectoderm into neural and non-neural fates^12^. Subsequently, the antagonistic interaction between the Bone Morphogenetic Protein and Wingless-related integration site pathways jointly induces and regulates the establishment of the prospective placodal ectoderm^13^. In the region of the otic placode, the dynamic coordination of fibroblast growth factor and Wingless-related integration site signaling induces the specialization of the prospective placodal ectoderm into the otic placode structure^14^. After the invagination of the otic placode forms the otic vesicle, neurogenesis is initiated by the expression of the proneural basic helix-loop-helix transcription factor Neurogenin 1, which is responsible for specifying the fate of neuronal progenitor cells and subsequently inducing the expression of another basic helix-loop-helix transcription factor, Neuronal Differentiation 1^15^. SOX2 is one of the earliest markers of the prospective sensory domain and is crucial for maintaining the undifferentiated state of neural progenitor cells in various developmental systems. The sequential upregulation of Neurogenin 1 and Neuronal Differentiation 1 in SOX2 cell subpopulations within the otic vesicle drives the differentiation of neuronal progenitors and neuroblasts, promoting the delamination of these cells from the otic vesicle and the formation of primary vestibular ganglia and spiral ganglion neurons^16^. SGNs are the main cell population of the cochlear ganglion, characterized by glutamatergic activity and a bipolar morphology, and can be subdivided into Type I (90%-95%) and Type II (5%-10%). Their peripheral processes precisely project to the bases and lateral walls of hair cells, forming ribbon synapses and receiving mechanoelectrical transduction signals; their central processes traverse the cochlear axis through the internal auditory canal, sequentially transmitting action potentials to the cochlear ventral nucleus and higher auditory centers, enabling efficient encoding and relay of sound information. Thus, SGNs play an indispensable hub role in maintaining normal auditory perception and verbal communication^17^, ^18^. The development of SGNs is cooperatively regulated by the neurotrophin family and various transcription factors. Among them, brain-derived neurotrophic factor (BDNF) and neurotrophin-3 (NT-3) control the survival, synaptogenesis, and axonal guidance of auditory neurons through activation of TrkB/TrkC receptors; transcription factors such as ISL1 and POU1F1 mediate neuronal migration, differentiation, and phenotypic specialization, together ensuring the precise integration of SGNs into the auditory pathway^19^-^21^.

### 2.2 Regulatory mechanisms of auditory nerve development

#### 2.2.1 Neurotrophic factor signaling axis

**Figure 1:**
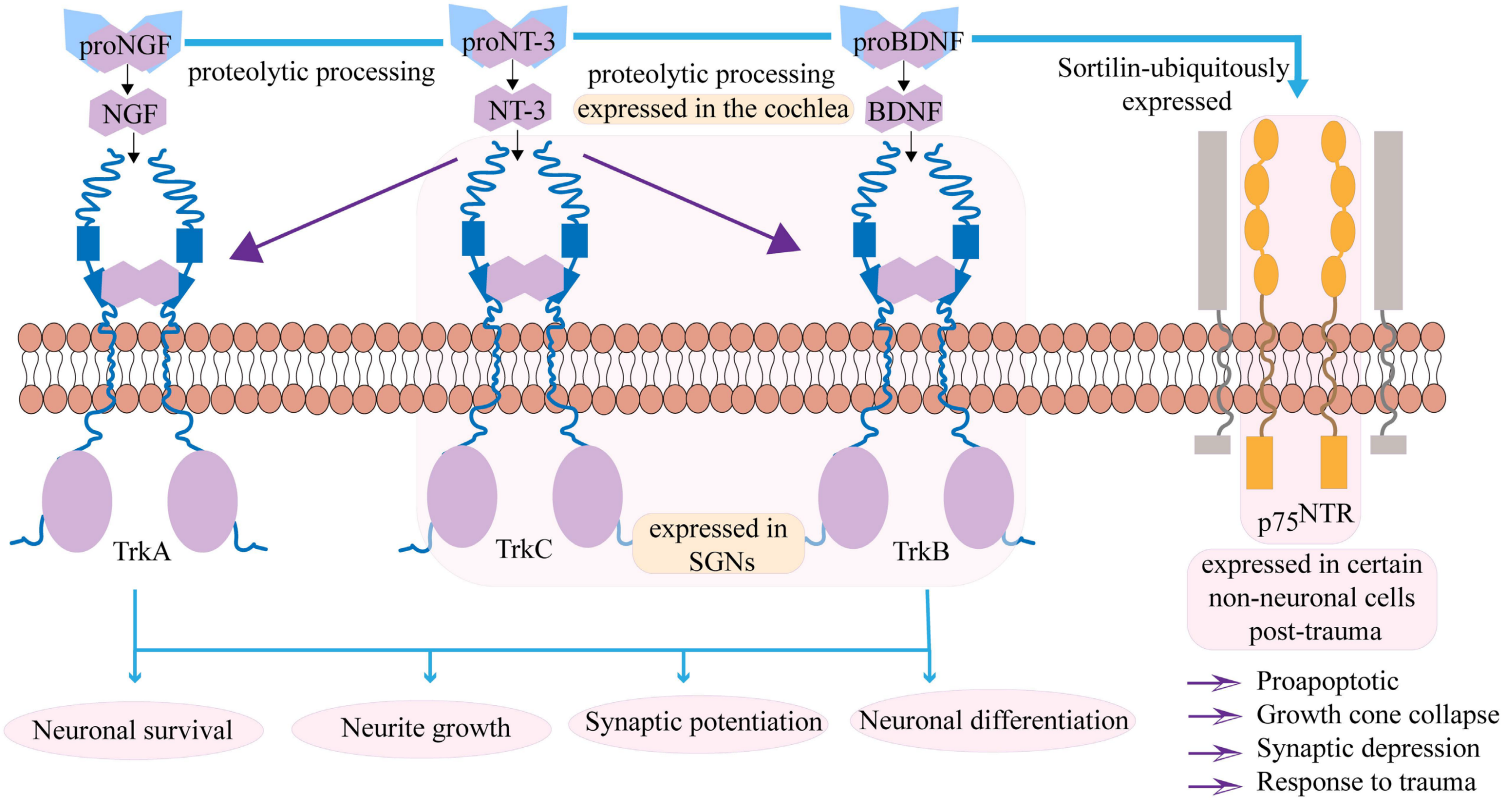
The neurotrophin family primarily includes BDNF, NT-3, and nerve growth factor. These factors are synthesized in their precursor forms and require cleavage by proteolytic enzymes to become mature, subsequently exerting their biological functions through binding to corresponding receptors. The interactions between neurotrophins and their receptors are regulated by a complex and precise cross-regulatory network. BDNF primarily binds to the tropomyosin receptor kinase B (TrkB), NGF selectively binds TrkA, while NT-3, in addition to its high-affinity receptor TrkC, can also bind to TrkA and TrkB. These interactions collectively regulate neuronal survival and differentiation, neurite outgrowth, and enhance synaptic plasticity. Both BDNF and NT-3 are expressed in the cochlea and SGNs. Mature neurotrophins can also bind to the p75NTR. When p75NTR is co-expressed with Trk receptors, it can significantly enhance the affinity and binding specificity of Trk receptors to their specific ligands, thereby achieving precise regulation of ligand-receptor interactions. Moreover, in certain non-neuronal cells following injury, the expression of p75NTR can contribute to the regulation of apoptosis, growth cone collapse, synaptic inhibition, and mediate responses to injury.

The neurotrophin family is a group of secreted proteins, including BDNF, nerve growth factor and NT-3, which are considered key regulators of nerve regeneration. Their biological functions are mediated by binding to specific transmembrane receptor systems: the tropomyosin receptor kinase family (TrkA, TrkB, TrkC) and the p75 neurotrophin receptor (p75NTR)^22^, ^23^. Nerve growth factor is synthesized as a precursor protein or pro-nerve growth factor and is processed at the N-terminal region (pro-domain) to produce the mature protein. Pro-nerve growth factor is released and is biologically active, mediating downstream signaling pathways^24^.The interactions between neurotrophins and their receptors are governed by a complex network of cross-regulatory mechanisms. The classic high-affinity binding paradigm involves specific neurotrophins binding to Trk receptors: BDNF and NT-4 bind to TrkB, NT-3 binds to TrkC, and NGF binds to TrkA. However, significant cross-binding occurs within this system. All mature neurotrophins can bind to the common receptor p75NTR, and this interaction plays a crucial regulatory role^25^-^27^, as shown in Figure 1. Notably, when p75NTR is co-expressed with Trk receptors, it can significantly enhance the affinity of Trk receptors for their specific ligands and improve binding specificity, enabling precise regulation of ligand-receptor interactions^28^. NT-3 primarily binds to the TrkC receptor, but it can also bind to TrkA and TrkB. The complexity of these interactions and their ultimate outcomes are further regulated by multiple factors, including neuronal activity, half-life, and the presence of co-receptors^29^.

**Figure 2:**
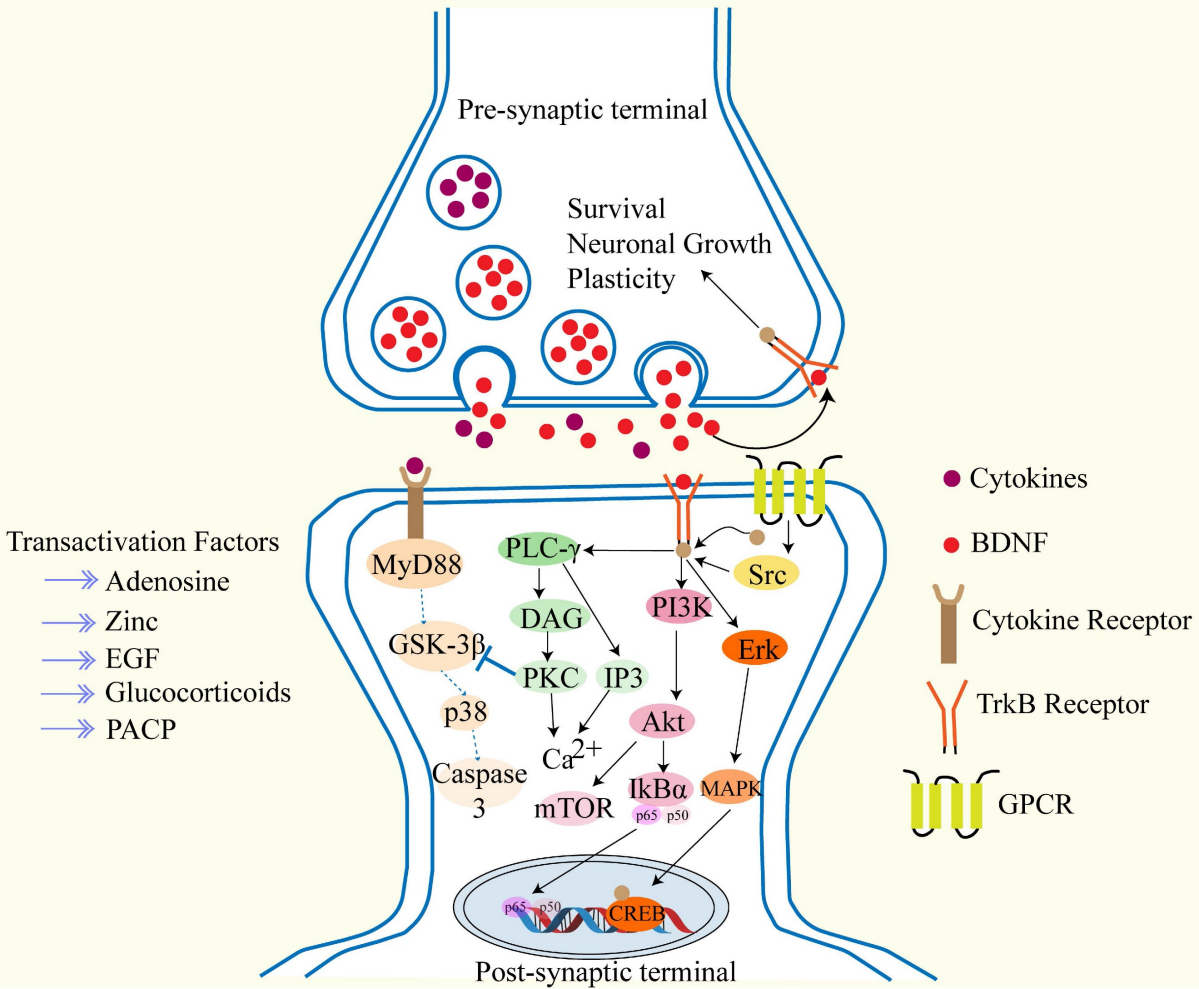
This figure shows that after BDNF binds to TrkB, it promotes neuronal growth and survival through three different signaling pathways.

During the development of the human fetal inner ear, the expression of TrkA, TrkB, TrkC, and p75NTR exhibits spatiotemporal specificity: TrkA begins to be expressed at the fifth gestational week and disappears after ninth gestational week; TrkB and TrkC reach their peak expression at eighth to twelfth gestational week, while p75NTR is persistently expressed throughout the entire developmental period^30^. BDNF binds to TrkB, and after phosphorylation through TrkB and the Phospho-Tyr785 site via phospholipase Cγ, it triggers Ca²⁺transients mediated by inositol 1,4,5-trisphosphate/diacylglycerol, which collectively drive synaptic plasticity through the following three cascade pathways: (1) activation of transient receptor potential canonical subfamily channels 3/6; (2) specific recruitment of α-amino-3-hydroxy-5-methyl-4-isoxazolepropionic acid receptor- GluR1 subunits to the synapse; (3) promotion of PSD-95-TrkB complex assembly via Ca²⁺/calmodulin-sensitive adenylyl cyclase and initiation of cyclic AMP response element-binding-dependent transcription^31^-^33^. After Shc-Grb2 binds to TrkB-Tyr515, it sequentially activates the Ras, Raf, MEK, and ERK pathways, phosphorylating CREB as well as eukaryotic initiation factor 4E, 4E-binding protein 1, and ribosomal protein S6, thereby coupling gene expression to protein synthesis-dependent plasticity. The Shc-Grb2 node can also recruit PI3K-Akt, which, by inhibiting the tuberous sclerosis complex proteins, relieves negative regulation of Rheb-mammalian target of rapamycin, drives translation mediated by p70S6K and 4E-BP1, and promotes membrane trafficking of synaptic proteins^34^-^36^. As shown in Figure 2. Current research confirms that BDNF and NT-3 and their receptors play a key regulatory role in the development and function of the inner ear, not only regulating the survival of spiral ganglion cells and neurons but also influencing nerve fiber growth, synaptic structure, and membrane physiological properties^37^. A study indicates^38^, that delivering BDNF to the inner ear via mesenchymal stem cell-derived small exosomes (sEVs) as nanocarriers can significantly reduce noise-induced cochlear hair cell loss in mice. In vitro studies have shown that both sEVs and BDNF-sEVs can directly protect hair cells against hydrogen peroxide-induced damage, with BDNF-sEVs exhibiting stronger synergistic protective effects, significantly alleviating oxidative stress, apoptosis, and nerve terminal degeneration. Another study^21^, reported that after inducing ototoxic damage in adult mice with neomycin, delivering BDNF and NT-3 to the mouse inner ear via an adenovirus vector derived from adenovirus type 28 increased the survival rate of spiral ganglion neurons. In summary, BDNF and NT-3 remodeling the synaptic connections between cochlear hair cells and SGNs has become one of the most promising biological strategies for treating SNHL.

#### 2.2.2 Regulation of transcription factors

Transcription factors are a class of DNA-binding proteins that can specifically recognize promoter or enhancer sequences upstream of genes and recruit or exclude RNA polymerase and its auxiliary complexes. In this way, they can precisely turn on or off the transcription of target genes, thereby determining cell fate, differentiation direction, and functional phenotype^39^. In the development of SGNs, a proneural transcription factor, Neurog1, is a key switch that initiates neurogenesis. In multipotent inner ear cells, Neurog1 enhances CDK2 levels to drive cell proliferation and promote neuronal differentiation by binding to its own promoter and the promoters of its target genes^40^. During the formation of the spiral ganglion and the establishment of peripheral and central auditory tonal expression, the transcription factor ISL1 is a core molecule that regulates the migration and pathfinding of SGNs. When ISL1 is lost, it leads to SGN migration defects, disorganized cochlear nerve distribution, and reduced or unseparated central axons^20^. The transcription factor Pou4f1 belongs to the POU transcription factor family and is broadly expressed in all SGNs starting from embryonic day 10^41^. Germline deletion of Pou4f1 causes early axon guidance errors in SGNs, preventing them from reaching central targets, which subsequently leads to neuronal apoptosis due to interruption of trophic signals, resulting in a permanent reduction of about 30% in adult cochlear neurons^42^. It is noteworthy that after hearing onset, Pou4f1 remains long-term in the cochlear apex-side type I SGNs, which can directly calibrate the voltage sensitivity and Ca²⁺ influx strength of presynaptic active zones, thereby maintaining high spontaneous and low-threshold hearing characteristics^43^. GATA3 belongs to the GATA transcription factor family in vertebrates and acts as a switch for the early identity of otic placode neuroblasts, primarily co-expressed in the anterior-ventral otic epithelium and corresponding neuroblasts. Gata3 is one of the earliest transcriptional markers in mouse inner ear development. In embryos with Gata3 deficiency, ear morphogenesis is blocked and inner ear structures are absent^44^, ^45^. In summary, transcription factors are key switches for the development of the inner ear and SGNs, controlling processes from otic placode formation, neuroblast identity determination, neuron proliferation and differentiation, cell migration, synapse targeting, to adjustment of calcium channel sensitivity after hearing onset. They precisely turn genes on or off throughout, ensuring the proper construction of auditory circuits. Therefore, activating or supplementing specific transcription factors through gene therapy or drugs holds the potential to reconstruct developmental programs and regenerate spiral ganglion neurons to restore their function, providing a new molecular entry point for precise intervention in SNHL.

## 3. Etiology and Pathogenesis of Auditory Nerve Injury

### 3.1 Auditory neuropathy spectrum disorder

Auditory neuropathy spectrum disorder (ANSD) is a relatively special type of sensorineural hearing loss. It is characterized by normal outer hair cell function, but abnormal function of inner hair cells, the stereocilia bundle, SGNs, and/or the auditory nerve itself, resulting in abnormal or even absent ABR waveforms, while distortion product otoacoustic emissions and cochlear sum potentials evoked by electrocochleography remain normal. Clinically, it can manifest as a disproportionately reduced speech recognition rate^46^-^48^. ANSD is mainly caused by genetic variations. Among these, mutations in the OTOF gene represent one of the most common genetic etiologies of non-syndromic ANSD. The OTOF gene, located on chromosome 2p23.3, belongs to the ferlin family of large transmembrane proteins and functions in regulating membrane fusion and vesicle formation^49^. This protein is selectively expressed in the inner and outer hair cells of the auditory system, vestibular receptors, and central nervous system tissues. Its core physiological mechanism lies in regulating the exocytosis of synaptic vesicles through calcium ion sensing, thereby facilitating mechano-electrical signal transduction^50^. Tsuzuki et al. found that OTOF gene-mutant mice exhibited a significant reduction in the number of spiral ganglion neurons type I (SGNS I), which was attributed to increased apoptosis, ultimately leading to the ANSD phenotype^51^. Notably, mutations in the OTOF gene are one of the major pathogenic factors for congenital deafness and autosomal recessive hereditary hearing loss, with clinical phenotypes characterized by severe to profound hearing impairment present at birth. The research results indicate that among infants with AN, the frequency of OTOF gene mutations is as high as 41.2%, suggesting that OTOF gene mutations are a major cause of congenital AN in Chinese infants^52^. Among the Japanese population, the prevalence is 56%, and among the Spanish population, OTOF mutations account for 87% of non-syndromic ANSD cases^53^, ^54^. Importantly, recent preclinical and clinical studies have demonstrated that bilateral OTOF gene therapy can restore hearing function in affected patients, with significant improvements in pure-tone thresholds and speech perception. This positions OTOF-related ANSD as an ideal candidate for precise gene replacement therapy and highlights the clinical significance of genetic diagnosis in selecting patients for such interventions. In addition to OTOF, studies have shown that mutations in the TMEM43 gene can lead to developmental defects in cochlear glial-like supporting cells and reduced gap junction functionality, thereby causing hearing impairment in ADSD patients^46^. Similarly, previous research has indicated that the AIFM1 c.1265 G > A variant is associated with ANSD, and its pathological mechanism is closely related to mitochondrial dysfunction and mCa²⁺ overload^55^. Thoroughly exploring the pathogenic mechanisms underlying the genetic heterogeneity of ANSD, including synaptic transmission defects (OTOF), supporting cell dysfunction (TMEM43), and mitochondrial metabolism (AIFM1), provides clear targets and a theoretical basis for precise gene therapy.

### 3.2 Noise-induced hearing loss

Noise-induced hearing loss (NIHL) refers to irreversible damage to the auditory system caused by a single intense impulse sound (such as an explosion or gunshot) or long-term repeated exposure to high-level noise environments. The main pathological sites are the outer hair cells of the cochlea and the spiral ganglion cells, leading to varying degrees of hearing loss^56^. Previous studies have shown that when people are exposed to 89 dB of sound for more than 5 hours per week, they may suffer permanent hearing damage over time^57^. The pathogenic factors of noise-induced hearing loss include many aspects, such as mechanical damage, metabolic damage, immune and inflammatory damage, and genetic susceptibility^58^. Generally, research suggests that when noise intensity is too high, sound waves are transmitted to the inner ear through the stapes, causing violent fluctuations between the perilymph and endolymph, resulting in strong shear forces between the basilar membrane and tectorial membrane. This leads to the breaking of tip links of the stereocilia and tearing of the cuticular plate, which can be observed as the destruction of outer hair cell stereocilia bundles^59^. Following metabolic overload, spasm and metabolic disorders of the cochlear capillaries occur, leading to excessive production of reactive oxygen species (ROS), triggering ischemia-reperfusion injury in the inner ear vasculature. The excessive ROS then begin to cause cellular damage, leading to protein denaturation and apoptosis of hair cells^60^. Meanwhile, noise exposure can rapidly trigger the simultaneous opening of voltage-dependent Ca^2+^ channels on hair cell membranes, generating inward calcium currents and causing a swift increase in intracellular free Ca^2+^ concentration. The sustained high Ca^2+^ signal not only damages mitochondria but, more importantly, high concentrations of Ca^2+^ activate calpains, which in turn further activate downstream proteases such as phospholipases, inducing lipid peroxidation and mitochondrial membrane permeabilization, ultimately initiating apoptotic pathways in hair cells. This is one of the key molecular pathways of noise-induced hearing loss^61^, ^62^. Within 1-2 days after noise exposure, the cochlea initiates an immune response, peaking with inflammation between 3-7 days, and then gradually subsiding, which clearly demonstrates that immune and inflammatory processes are deeply involved in and promote the progression of noise-induced hearing loss^63^. In addition to hair cell loss, recent evidence suggests that noise exposure can directly damage auditory nerve fibers and their synaptic connections with inner hair cells (IHCs). This cochlear synaptopathy, often referred to as “hidden hearing loss”, involves the loss of ribbon synapses between IHCs and type I SGN afferent fibers, even when hair cells remain morphologically intact. Excessive noise exposure induces glutamate excitotoxicity, causing excessive Ca^2+^ influx through postsynaptic AMPA receptors, triggering calpain activation, and subsequently dismantling presynaptic ribbon structures and postsynaptic densities^64^. As a result, the auditory nerve undergoes primary degenerative changes characterized by axonal retraction, demyelination of fibers in the bony spiral lamina, and delayed loss of SGN cell bodies in Rosenthal's canal^65^. Notably, this neuropathy can occur at noise levels below those at which threshold shifts are detectable by standard hearing tests. However, due to reduced auditory nerve firing precision and increased spontaneous firing rates, it leads to deficits in temporal processing and speech perception in noise^66^. Moreover, noise-induced oxidative stress and mitochondrial dysfunction may retrogradely spread from the cochlea to the cochlear nucleus and superior olivary complex, resulting in increased central gain and maladaptive neural reorganization, thereby worsening tinnitus and sound hypersensitivity^67^. The extent of hearing damage caused by noise is not determined solely by sound intensity; individual differences can exceed 10 dB. Genetic susceptibility also plays a crucial role. For example, mutations in ABCC1, part of the ATP-binding cassette transporter superfamily, can reduce the cochlea's antioxidant capacity, thereby amplifying noise-induced oxidative stress and hearing loss^68^. Similarly, CDH23, located at the lateral links of stereocilia during development, not only assists in shaping the hair bundle but also ensures efficient transmission of mechanical force to the transduction channels. Additionally, the polymorphic site rs3802711 in CDH13 (BB genotype and B allele) is significantly associated with susceptibility to acquired noise-induced hearing loss^69^. In summary, noise-induced hearing loss is a complex disorder involving physical, metabolic, immune-inflammatory, and genetic factors. Mechanical energy triggers calcium overload and oxidative stress, while immune-inflammatory responses and related genetic susceptibility factors collectively determine individual outcomes, providing predictable and targetable molecular targets for precision intervention.

### 3.3 Age-related degeneration

Presbycusis, also known as age-related hearing loss, refers to the progressive bilateral degenerative hearing decline that occurs with aging and is the most common cause of hearing impairment in adults^70^, ^71^. In individuals aged 65 and older, approximately 33% experience severe hearing impairment, primarily manifested as reduced speech recognition sensitivity in noisy environments, diminished central auditory processing capacity and speed, and difficulty with sound localization. Severe presbycusis can even lead to cognitive issues such as loneliness, depression, and dementia^72^. Among its subtypes, sensorineural presbycusis is a prominent one, characterized pathologically by a reduction in spiral ganglion neuron numbers and thinning of axonal myelin, while outer hair cells and the basilar membrane remain largely intact in the early to middle stages^73^. The loss of inner hair cells and auditory nerve fiber projections is considered a critical early event in sensorineural presbycusis^74^. Clinical studies have found that in age-related hearing loss, suprathreshold auditory nerve responses are reduced, while the thinning of the temporal-parietal cortex is positively correlated with processing speed. This suggests that the decline in peripheral SGN function is directly related to central atrophy and cognitive decline^75^. At the molecular level, mitochondrial function is closely associated with hearing loss caused by degenerative changes in the auditory system. For example, in the aged cochlea with auditory degeneration, FOXG1 plays a crucial role through autophagy. Inhibition of FOXG1 reduces autophagic activity, leading to the accumulation of ROS and apoptosis of cochlear hair cells^76^. Single-cell analysis further revealed that decreased expression of the transmembrane transporter SLC35F1 causes degeneration of cochlear hair cells and progressive decline in auditory function^77^. Additionally, in a d-galactose-induced aging model, iron deposition was observed in the auditory cortex, accompanied by increased iron-regulatory protein IRP-2 and elevated lipid peroxidation marker MDA, which leads to deformation of auditory nerve ganglion neurons, suggesting that ferroptosis is involved in the degeneration of central auditory neurons^78^. In short, a key component of hearing loss caused by age-related degeneration lies in the deterioration of the auditory nerve, and factors such as mitochondria, transport membrane proteins, and metabolic disorders play important roles in this process. Targeting these aspects can effectively delay SGN degeneration, providing a practical molecular approach for the precise prevention and treatment of age-related hearing loss.

### 3.4 Other injuries such as ototoxicity

**Figure 3:**
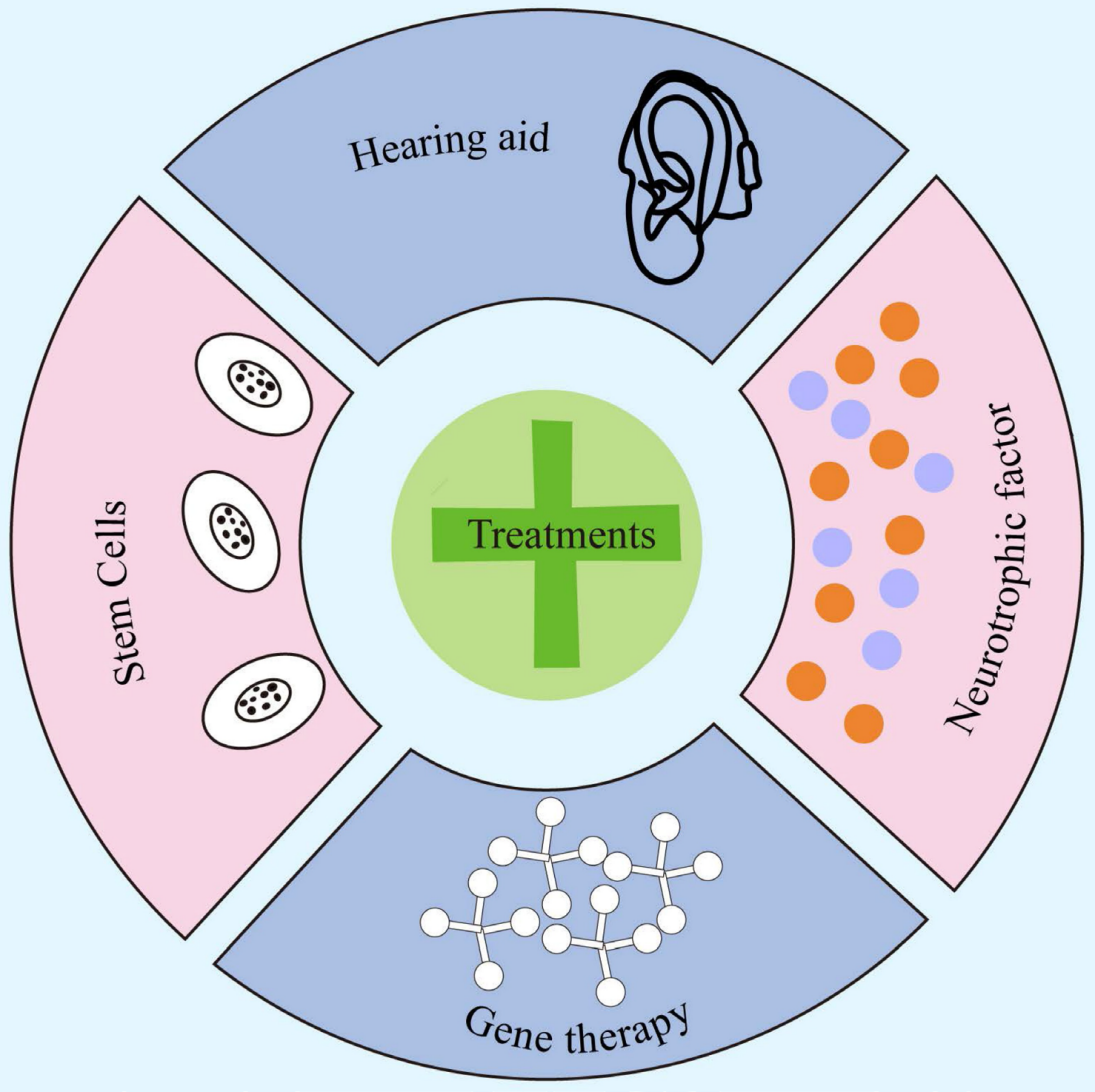
Several causes can lead to auditory nerve damage, resulting in hearing loss.

Ototoxicity refers to dose-dependent structural damage and functional impairment to the cochlea, vestibular system, or auditory central structures caused by exogenous factors such as drugs, chemical agents, or heavy metals. As shown in Figure 3, the main clinical manifestations are hearing loss, tinnitus, vertigo, or balance disorders. The damage can affect outer cochlear hair cells, SGNs, stria vascularis, and vestibular cells^79^, ^80^, and is often irreversible. Clinically, aminoglycoside antibiotics are among the most classic ototoxic drugs. They mainly catalyze a burst of ROS in cochlear cells by forming iron-amino complexes, and can also damage spiral ganglion neurons, causing bilateral high-frequency onset, permanent sensorineural hearing loss, and often temporary vestibular function decline^81^, ^82^. Cisplatin is a commonly used platinum-based chemotherapeutic drug in clinical settings, and sensorineural hearing loss is one of its dose-limiting side effects. Once the drug enters the inner ear, it rapidly accumulates in cells, causing damage to cochlear hair cell organelle metabolism, inducing oxidative stress and ferroptosis, while simultaneously activating the ATM-Chk2-p53 pathway to exert a decisive effect^83^. Cisplatin-induced ototoxicity involves multiple programmed cell death pathways, including apoptosis, necroptosis, autophagy, and ferroptosis. This results in cisplatin-induced ototoxicity exhibiting multi-target and multi-modal characteristics, making it difficult for traditional single-pathway inhibitors to provide complete protection, which is a major challenge in clinical prevention and treatment^84^. Besides commonly used drugs, organic solvents and heavy metals are also clinically significant ototoxic factors, capable of causing severe damage to cochlear structures and hearing function. Industrial aromatic hydrocarbon solvents, represented by toluene, can cause loss of outer hair cells in the middle and basal turns of the organ of Corti^85^. Continuous exposure can lead to a significant elevation in BAER thresholds^86^. Exposure to styrene can result in hearing loss of approximately 35-40 dB and simultaneously induce oxidative-reductive and inflammatory responses, targeting hair cell and spiral ganglion neuron dysfunction through oxidation and inflammation^87^. Studies have shown that heavy metals such as arsenic, lead, and mercury may have synergistic effects with noise, with lead being particularly harmful, causing impairments in both peripheral and central auditory function^88^. A meta-analysis covering 10 studies indicated that there is an association between lead exposure and hearing loss, with the risk of hearing loss positively correlated with high concentrations of lead exposure^89^. Whether it is the oxidative and inflammatory responses induced by industrial solvents, or the potential synergistic damage from heavy metals and noise, these findings suggest that ototoxicity is not simply a classical paradigm of drugs acting on a single target, but rather a multi-damage model determined by neurodevelopment, metabolic oxidative imbalance, and cochlear structural damage. Therefore, prevention and treatment strategies must shift from single antagonism to multi-pathway collaboration to provide a precise direction for reducing ototoxicity.

### 3.5 Human cytomegalovirus infection

Human cytomegalovirus (CMV), rubella virus, herpes simplex virus, and human immunodeficiency virus can all cause sensorineural hearing loss^90^. Among these, CMV is the primary viral cause of congenital deafness^91^. The possible mechanisms are as follows: on one hand, CMV increases the expression of ROS, which can activate the NOD-like receptor family pyrin domain-containing 3 inflammasome in SGNs, leading to the activation of Caspase 1 and enhancing the maturation and release of IL-1β and IL-18, thereby mediating inflammatory responses; on the other hand, CMV can induce a sustained increase in Ca^2+^, resulting in SGN apoptosis^92^-^94^. Cohort study data indicate that infants with postnatal CMV infection exhibit a higher rate of failing otoacoustic emission screening^95^. Chatzakis^96^ et al. reported that in cases of fetal infection, CMV DNA can be detected via amniocentesis, with a 22% probability of severe symptoms. Conducting hearing screening and antiviral treatment for newborns can reduce the incidence of SNHL following CMV infection. CMV can damage SGNs, and further research is needed to elucidate the mechanisms underlying SGN injury, providing a potential new strategy for the treatment of SNHL.

## 4. Therapeutic Strategies and Prospects Targeting the Auditory Nerve

### 4.1 Strategies for neuroprotection and regeneration

#### 4.1.1 Neurotrophic factor therapy

Neurotrophic factors (NTFs) include the classic neurotrophic protein family and neuropeptides. They are secreted proteins that regulate the survival, development, and plasticity of neurons in the central and peripheral nervous systems. They can maintain normal neuronal activity and also initiate repair signals after injuries such as trauma, repair, and neurodegeneration^97^. In the auditory system, NTFs play a key role in the development of SGNs. When cochlear implants are used, the quantity and quality of SGNs, as the target cells for the implant, directly affect the effectiveness of the cochlear implant^98^. Within the neurotrophic factor family, NT-3 in particular has been shown to repair damaged SGNs and restore synapses in the auditory system; therefore, it is regarded as a key molecular enhancer for cochlear implant performance^99^. Likewise, neurotrophic factors are crucial during SGN development. Animal studies have shown that mice lacking the NT-3 gene or its receptor TrkC experience significant loss of spiral ganglion cells in the cochlear basal turn and complete loss of innervation in the inner ear^100^. However, due to the special structure of the ear, clinical application of neurotrophic factors requires addressing issues related to drug delivery and treatment protocols. NTFs in solution can be injected into the cochlea via cochleostomy through the cochlear bony wall or round window membrane^101^, but this inevitably causes a certain degree of damage. Recent studies on minimally invasive delivery have shown that PLGA temperature-sensitive hydrogels loaded with brain-derived neurotrophic factor can reverse synaptopathy and hearing loss in NIHL mouse models^102^. Additionally, viral-mediated NT-3 cochlear gene therapy has also significantly improved damage and reduced abnormal SGN morphology in rodent models^103^. In summary, from surgical perfusion to hydrogel slow release, from protein supplementation to gene therapy, neurotrophic factors provide a precise, minimally invasive, and feasible intervention pathway for repairing SGN damage and reversing disease in sensorineural hearing loss.

#### 4.1.2 Gene therapy and stem cell therapy

**Figure 4:**
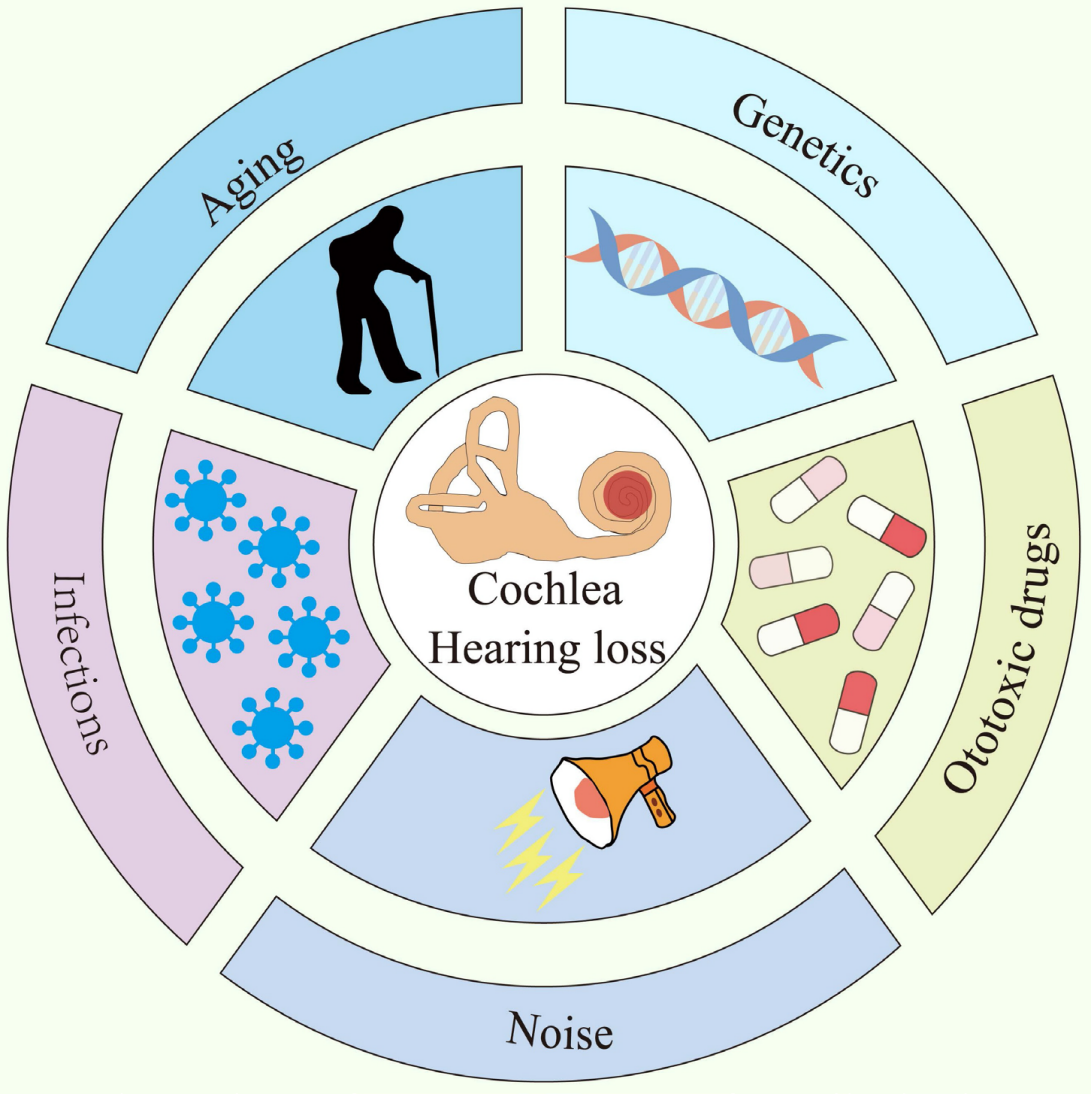
Comprehensive therapeutic strategies and prospects targeting the auditory nerve: focusing on neuroprotection, regulation of the regenerative microenvironment, delivery of neurotrophic factors, synergistic repair via gene editing/stem cell transplantation, and innovations in artificial auditory reconstruction technologies. These strategies aim to surpass the limitations of traditional cochlear implants and envision the clinical translation of emerging technologies such as optogenetics, ultrasound, flexible electrodes, and organoid-based approaches.

In addition to the neurotrophic factor therapies mentioned above, gene therapy and stem cell therapy have also been extensively studied and applied in the field of sensorineural hearing loss. As shown in Figure 4. Gene therapy primarily uses viral vectors, such as adeno-associated viruses or lentiviruses, to deliver therapeutic genes targeted to the inner ear. This approach is suitable for treating recessive genetic disorders like autosomal recessive DFNB9 caused by OTOF mutations, as well as dominant genetic disorders resulting from haploinsufficiency of a single allele^104^. Gene therapy for DFNB9 has been validated for feasibility in mouse models and is expected to further advance related treatments^105^. Since this type of cellular damage is permanent, regenerative treatment strategies have been explored, leading to the development of stem cell-based regenerative therapies^106^. Stem cell therapy involves differentiating embryonic stem cells, adult stem cells, or induced pluripotent stem cells in vitro into cells with auditory neuron characteristics, which are then transplanted into the damaged cochlea to repair SGNs or cochlear hair cell injuries caused by noise, aging, ototoxic drugs, or genetic factors, thereby reconstructing the auditory conduction pathway and restoring or improving hearing function^107^, ^108^. The differentiation efficiency of stem cells into hair cells and auditory neurons is remarkably high, with hair cell differentiation success rates reaching 82% and auditory neurons nearly 100%. Moreover, these differentiated cells can form new synapses and interact with surrounding cochlear explant cells and neurons, showing promising therapeutic potential. However, this therapy still faces many challenges in the preclinical stage, including limited ethical approvals, destruction during embryo separation, and immune rejection following transplantation^106^. Gene therapy and stem cell therapy constitute two complementary strategies in regenerative medicine, providing transformative intervention frameworks for sensorineural hearing loss from the perspectives of gene correction and cell replacement, respectively. Although clinical translation from the laboratory remains limited by constraints such as pre-existing vectors, stem cell sources, and post-transplant functional integration, these studies have already established a clear research pathway for the regeneration of auditory function.

### 4.2 Innovations in strategies for artificial auditory reconstruction

#### 4.2.1 Limitations and optimization of traditional cochlear implants

Cochlear implants are implantable neural prosthetic devices that restore sensory functions in humans and essentially act as electronic substitutes for sensory hair cells. As of 2012, 324,000 patients worldwide had received cochlear implants, successfully restoring auditory perception in individuals with severe to profound hearing loss^109^. However, cochlear implants still face several limitations: patients may have restrictions during postoperative MRI examinations due to magnetic artifacts or thermal effects^110^. Speech understanding in noisy environments remains difficult, likely due to inadequate low-frequency resolution caused by broad current spread from the stimulation sites^111^. Moreover, complications such as meningitis, infections, cerebrospinal fluid leaks, electrode extrusion, and facial nerve stimulation may occur after cochlear implantation, with incidence rates related to both surgeon experience and implant design^112^. Despite these challenges, cochlear implant technology continues to advance. For example, the use of new Spectral Peak and Continuous Interleaved Sampling strategies has significantly improved speech recognition performance^113^. Notably, neurotrophic factor-assisted therapies can enhance cochlear implant outcomes and improve the survival of SGNs^98^.

#### 4.2.2 Prospects for emerging technologies

For sensorineural hearing loss, in addition to traditional cochlear implants, several emerging technologies are gradually moving from the laboratory to clinical use. Auditory brainstem implants were initially used to restore auditory perception in patients with type 2 neurofibromatosis deafness and are now being expanded to treat children with congenital deafness due to cochlear malformations and cochlear nerve deficiency^114^. Optogenetic cochlear implants are considered an important method for hearing restoration. They address the limitations of traditional cochlear implant electrical stimulation in conductive environments like the cochlea, where sound encoding precision and hearing quality are restricted. By using light stimulation, they achieve higher spectral selectivity and can precisely activate auditory nerves over a smaller tonal range, significantly improving hearing quality^115^. Additionally, stem cell-based strategies are actively being explored. By inducing pluripotent stem cells to differentiate into functional auditory neurons and implanting them into the auditory pathway, it is possible to rebuild synaptic input-output circuits and achieve fundamental regeneration of neural structures^116^. From the expansion of ABI indications and the precise encoding of optogenetics to structural regeneration using stem cells, these emerging technologies together offer a new pathway for treating sensorineural hearing loss.

## 5. Conclusion

The auditory nervous system, SGNs plays an irreplaceable and central role in maintaining normal auditory function. As the sole bridge connecting cochlear hair cells to central auditory pathways, SGNs are not only responsible for converting mechanical sound signals into electrochemical nerve impulses but also carry the crucial mission of ensuring the accurate transmission of auditory information. When SGNs undergo degenerative changes, this delicate auditory conduction pathway suffers serious disruption, leading to a sharp decline in hearing, or even complete loss. Future research should further explore the specific molecular mechanisms underlying SGN damage, develop more precise neuroprotective strategies, optimize stem cell transplantation and neurotrophic factor delivery techniques, and establish personalized treatment plans. With the rapid advancement of single-cell sequencing technology, gene editing, and biomaterials science, precision medical treatments based on SGN regeneration and protection will bring new hope to patients with hearing impairments.

## References

[1] Chadha S, Kamenov K, Cieza A (2021). The world report on hearing, 2021. Bull World Health Organ.

[2] Ono M, Ito T (2024). Hearing loss-related altered neuronal activity in the inferior colliculus. Hear Res.

[3] Tan W, Song L (2023). Role of mitochondrial dysfunction and oxidative stress in sensorineural hearing loss. Hear Res.

[4] Wan H, Zhang Y, Hua Q (2022). Cellular autophagy, the compelling roles in hearing function and dysfunction. Front Cell Neurosci.

[5] Wu Y, Zhang J, Liu Q, Miao Z, Chai R, Chen W (2024). Development of Chinese herbal medicine for sensorineural hearing loss. Acta Pharm Sin B.

[6] Wang J, Zheng J, Wang H, He H, Li S, Zhang Y (2023). Gene therapy: an emerging therapy for hair cells regeneration in the cochlea. Front Neurosci.

[7] Quimby AE, Wei K, Adewole D, Eliades S, Cullen DK, Brant JA (2023). Signal processing and stimulation potential within the ascending auditory pathway: a review. Front Neurosci.

[8] Zine A, Messat Y, Fritzsch B (2021). A human induced pluripotent stem cell-based modular platform to challenge sensorineural hearing loss. Stem Cells.

[9] Zhang L, Chen S, Sun Y (2021). Mechanism and Prevention of Spiral Ganglion Neuron Degeneration in the Cochlea. Front Cell Neurosci.

[10] Li Z, Gao Y, Chen X, Xu L, Li Z, Chai R (2025). Study on Recovery Strategy of Hearing Loss & SGN Regeneration Under Physical Regulation. Adv Sci (Weinh).

[11] Pavlinkova G (2020). Molecular Aspects of the Development and Function of Auditory Neurons. Int J Mol Sci.

[12] De Robertis EM, Kuroda H (2004). Dorsal-ventral patterning and neural induction in Xenopus embryos. Annu Rev Cell Dev Biol.

[13] Tambalo M, Anwar M, Ahmed M, Streit A (2020). Enhancer activation by FGF signalling during otic induction. Dev Biol.

[14] Singh S, Groves AK (2016). The molecular basis of craniofacial placode development. Wiley Interdiscip Rev Dev Biol.

[15] Liu M, Pereira FA, Price SD, Chu MJ, Shope C, Himes D (2000). Essential role of BETA2/NeuroD1 in development of the vestibular and auditory systems. Genes Dev.

[16] Evsen L, Sugahara S, Uchikawa M, Kondoh H, Wu DK (2013). Progression of neurogenesis in the inner ear requires inhibition of Sox2 transcription by neurogenin1 and neurod1. J Neurosci.

[17] Liu W, Xu X, Fan Z, Sun G, Han Y, Zhang D (2019). Wnt Signaling Activates TP53-Induced Glycolysis and Apoptosis Regulator and Protects Against Cisplatin-Induced Spiral Ganglion Neuron Damage in the Mouse Cochlea. Antioxid Redox Signal.

[18] Sun S, Babola T, Pregernig G, So KS, Nguyen M, Su SM (2018). Hair Cell Mechanotransduction Regulates Spontaneous Activity and Spiral Ganglion Subtype Specification in the Auditory System. Cell.

[19] Kempfle JS, Duro MV, Zhang A, Amador CD, Kuang R, Lu R (2021). A Novel Small Molecule Neurotrophin-3 Analogue Promotes Inner Ear Neurite Outgrowth and Synaptogenesis In vitro. Front Cell Neurosci.

[20] Filova I, Pysanenko K, Tavakoli M, Vochyanova S, Dvorakova M, Bohuslavova R (2022). ISL1 is necessary for auditory neuron development and contributes toward tonotopic organization. Proc Natl Acad Sci U S A.

[21] St PM, Brough DE, Lawrence A, Nelson-Brantley J, Huang P, Harre J (2022). Improving Control of Gene Therapy-Based Neurotrophin Delivery for Inner Ear Applications. Front Bioeng Biotechnol.

[22] Chao MV, Hempstead BL (1995). p75 and Trk: a two-receptor system. Trends Neurosci.

[23] Fritzsch B, Tessarollo L, Coppola E, Reichardt LF (2004). Neurotrophins in the ear: their roles in sensory neuron survival and fiber guidance. Prog Brain Res.

[24] Lee R, Kermani P, Teng KK, Hempstead BL (2001). Regulation of cell survival by secreted proneurotrophins. Science.

[25] Klein R, Nanduri V, Jing SA, Lamballe F, Tapley P, Bryant S (1991). The trkB tyrosine protein kinase is a receptor for brain-derived neurotrophic factor and neurotrophin-3. Cell.

[26] Benedetti M, Levi A, Chao MV (1993). Differential expression of nerve growth factor receptors leads to altered binding affinity and neurotrophin responsiveness. Proc Natl Acad Sci U S A.

[27] Keefe KM, Sheikh IS, Smith GM (2017). Targeting Neurotrophins to Specific Populations of Neurons: NGF, BDNF, and NT-3 and Their Relevance for Treatment of Spinal Cord Injury. Int J Mol Sci.

[28] Bibel M, Hoppe E, Barde YA (1999). Biochemical and functional interactions between the neurotrophin receptors trk and p75NTR. EMBO J.

[29] Yano H, Torkin R, Martin LA, Chao MV, Teng KK (2009). Proneurotrophin-3 is a neuronal apoptotic ligand: evidence for retrograde-directed cell killing. J Neurosci.

[30] Steinacher C, Nishio SY, Usami SI, Dudas J, Rieder D, Rask-Andersen H (2024). Expression of Neurotrophins and Its Receptors During Fetal Development in the Human Cochlea. Int J Mol Sci.

[31] Amaral MD, Pozzo-Miller L (2007). TRPC3 channels are necessary for brain-derived neurotrophic factor to activate a nonselective cationic current and to induce dendritic spine formation. The Journal of neuroscience: the official journal of the Society for Neuroscience.

[32] Ji Y, Pang PT, Feng L, Lu B (2005). Cyclic AMP controls BDNF-induced TrkB phosphorylation and dendritic spine formation in mature hippocampal neurons. Nat Neurosci.

[33] Shaywitz AJ, Greenberg ME (1999). CREB: a stimulus-induced transcription factor activated by a diverse array of extracellular signals. Annu Rev Biochem.

[34] Huang EJ, Reichardt LF (2003). Trk receptors: roles in neuronal signal transduction. Annu Rev Biochem.

[35] Yoshii A, Constantine-Paton M (2007). BDNF induces transport of PSD-95 to dendrites through PI3K-AKT signaling after NMDA receptor activation. Nat Neurosci.

[36] Yoshii A, Constantine-Paton M (2010). Postsynaptic BDNF-TrkB signaling in synapse maturation, plasticity, and disease. Dev Neurobiol.

[37] Green SH, Bailey E, Wang Q, Davis RL (2012). The Trk A, B, C's of neurotrophins in the cochlea. Anat Rec (Hoboken).

[38] Min X, Deng XH, Lao H, Wu ZC, Chen Y, Luo Y (2023). BDNF-enriched small extracellular vesicles protect against noise-induced hearing loss in mice. J Control Release.

[39] Hsia CC, McGinnis W (2003). Evolution of transcription factor function. Curr Opin Genet Dev.

[40] Song Z, Jadali A, Fritzsch B, Kwan KY (2017). NEUROG1 Regulates CDK2 to Promote Proliferation in Otic Progenitors. Stem Cell Reports.

[41] Deng M, Yang H, Xie X, Liang G, Gan L (2014). Comparative expression analysis of POU4F1, POU4F2 and ISL1 in developing mouse cochleovestibular ganglion neurons. Gene expression patterns: GEP.

[42] Huang EJ, Liu W, Fritzsch B, Bianchi LM, Reichardt LF, Xiang M (2001). Brn3a is a transcriptional regulator of soma size, target field innervation and axon pathfinding of inner ear sensory neurons. Development (Cambridge, England).

[43] Sherrill HE, Jean P, Driver EC, Sanders TR, Fitzgerald TS, Moser T (2019). Pou4f1 Defines a Subgroup of Type I Spiral Ganglion Neurons and Is Necessary for Normal Inner Hair Cell Presynaptic Ca(2+) Signaling. The Journal of neuroscience: the official journal of the Society for Neuroscience.

[44] Lilleväli K, Matilainen T, Karis A, Salminen M (2004). Partially overlapping expression of Gata2 and Gata3 during inner ear development. Developmental dynamics: an official publication of the American Association of Anatomists.

[45] Nishimura K, Noda T, Dabdoub A (2017). Dynamic Expression of Sox2, Gata3, and Prox1 during Primary Auditory Neuron Development in the Mammalian Cochlea. PLoS One.

[46] Kang X, Ma L, Wen J, Gong W, Liu X, Hu Y (2025). Modeling of auditory neuropathy spectrum disorders associated with the TEME43 variant reveals impaired gap junction function of iPSC-derived glia-like support cells. Front Mol Neurosci.

[47] Kaga K (2016). Auditory nerve disease and auditory neuropathy spectrum disorders. Auris, nasus, larynx.

[48] Huang Y, Yang J, Duan M (2022). Auditory neuropathy: from etiology to management. Curr Opin Otolaryngol Head Neck Surg.

[49] Xue Y, Tao Y, Wang X, Wang X, Shu Y, Liu Y (2023). RNA base editing therapy cures hearing loss induced by OTOF gene mutation. Molecular therapy: the journal of the American Society of Gene Therapy.

[50] Yang J, Zhang D, Wang K (2025). Pathogenesis and research progress of OTOF gene related auditory neuropathy: a retrospective review. Am J Transl Res.

[51] Tsuzuki N, Namba K, Saegusa C, Mutai H, Nishiyama T, Oishi N (2023). Apoptosis of type I spiral ganglion neuron cells in Otof-mutant mice. Neurosci Lett.

[52] Zhang Q, Han B, Lan L, Zong L, Shi W, Wang H (2016). High frequency of OTOF mutations in Chinese infants with congenital auditory neuropathy spectrum disorder. Clin Genet.

[53] Rodríguez-Ballesteros M, Reynoso R, Olarte M, Villamar M, Morera C, Santarelli R (2008). A multicenter study on the prevalence and spectrum of mutations in the otoferlin gene (OTOF) in subjects with nonsyndromic hearing impairment and auditory neuropathy. Hum Mutat.

[54] Matsunaga T, Mutai H, Kunishima S, Namba K, Morimoto N, Shinjo Y (2012). A prevalent founder mutation and genotype-phenotype correlations of OTOF in Japanese patients with auditory neuropathy. Clin Genet.

[55] Qiu Y, Wang H, Fan M, Pan H, Guan J, Jiang Y (2023). Impaired AIF-CHCHD4 interaction and mitochondrial calcium overload contribute to auditory neuropathy spectrum disorder in patient-iPSC-derived neurons with AIFM1 variant. Cell Death Dis.

[56] Ding T, Yan A, Liu K (2019). What is noise-induced hearing loss?. British journal of hospital medicine (London, England: 2005).

[57] Imam L, Hannan SA (2017). Noise-induced hearing loss: a modern epidemic?. British journal of hospital medicine (London, England: 2005).

[58] Chen K, Su S, Chen K (2020). An overview of occupational noise-induced hearing loss among workers: epidemiology, pathogenesis, and preventive measures. Environ Health Prev Med.

[59] Van Eynde C, Denys S, Desloovere C, Wouters J, Verhaert N (2016). Speech-in-noise testing as a marker for noise-induced hearing loss and tinnitus. B-ENT.

[60] Kurabi A, Keithley EM, Housley GD, Ryan AF, Wong AC (2017). Cellular mechanisms of noise-induced hearing loss. Hear Res.

[61] Yamaguchi T, Yoneyama M, Ogita K (2017). Calpain inhibitor alleviates permanent hearing loss induced by intense noise by preventing disruption of gap junction-mediated intercellular communication in the cochlear spiral ligament. Eur J Pharmacol.

[62] Minami SB, Yamashita D, Schacht J, Miller JM (2004). Calcineurin activation contributes to noise-induced hearing loss. J Neurosci Res.

[63] Wood MB, Zuo J (2017). The Contribution of Immune Infiltrates to Ototoxicity and Cochlear Hair Cell Loss. Front Cell Neurosci.

[64] Suthakar K, Liberman MC (2021). Auditory-nerve responses in mice with noise-induced cochlear synaptopathy. J Neurophysiol.

[65] Kujawa SG, Liberman MC (2009). Adding insult to injury: cochlear nerve degeneration after "temporary" noise-induced hearing loss. The Journal of neuroscience: the official journal of the Society for Neuroscience.

[66] Kujawa SG, Liberman MC (2015). Synaptopathy in the noise-exposed and aging cochlea: Primary neural degeneration in acquired sensorineural hearing loss. Hear Res.

[67] Tagoe T, Barker M, Jones A, Allcock N, Hamann M (2014). Auditory nerve perinodal dysmyelination in noise-induced hearing loss. The Journal of neuroscience: the official journal of the Society for Neuroscience.

[68] Liu J, Bai Y, Feng Y, Liu X, Pang B, Zhang S (2024). ABCC1 deficiency potentiated noise-induced hearing loss in mice by impairing cochlear antioxidant capacity. Redox Biol.

[69] Chen K, Huang B, Sun J, Liang Y, Xiong G (2022). Cochlear Implantation Outcomes in Children With CDH23 Mutations-Associated Hearing Loss. Otolaryngology-head and neck surgery: official journal of American Academy of Otolaryngology-Head and Neck Surgery.

[70] Gates GA, Mills JH (2005). Presbycusis. Lancet (London, England).

[71] Zhao X, Shen T, Cao S, Liu Z, Pang W, Li M (2025). Presbycusis: Pathology, Signal Pathways, and Therapeutic Strategy. Advanced science (Weinheim, Baden-Wurttemberg, Germany).

[72] Sun Z, Cheng Z, Gong N, Xu Z, Jin C, Wu H (2021). Neural presbycusis at ultra-high frequency in aged common marmosets and rhesus monkeys. Aging.

[73] Nelson EG, Hinojosa R (2006). Presbycusis: a human temporal bone study of individuals with downward sloping audiometric patterns of hearing loss and review of the literature. The Laryngoscope.

[74] Steenken F, Heeringa AN, Beutelmann R, Zhang L, Bovee S, Klump GM (2021). Age-related decline in cochlear ribbon synapses and its relation to different metrics of auditory-nerve activity. Neurobiol Aging.

[75] Delano PH, Belkhiria C, Vergara RC, Martínez M, Leiva A, Andrade M (2020). Reduced suprathreshold auditory nerve responses are associated with slower processing speed and thinner temporal and parietal cortex in presbycusis. PLoS One.

[76] He Z, Li M, Fang Q, Liao F, Zou S, Wu X (2021). FOXG1 promotes aging inner ear hair cell survival through activation of the autophagy pathway. Autophagy.

[77] Sun G, Fu X, Zheng Y, Hong G, Liu Z, Luo B (2025). Single-cell profiling identifies hair cell SLC35F1 deficiency as a signature of primate cochlear aging. Nat Aging.

[78] Chen X, Li D, Sun H, Wang W, Wu H, Kong W (2020). Relieving ferroptosis may partially reverse neurodegeneration of the auditory cortex. The FEBS journal.

[79] Steyger PS (2021). Mechanisms of Ototoxicity and Otoprotection. Otolaryngol Clin North Am.

[80] Naples JG, Rice-Narusch W, Watson NW, Ghulam-Smith M, Holmes S, Li D (2023). Ototoxicity Review: A Growing Number of Non-Platinum-Based Chemo- and Immunotherapies. Otolaryngology-head and neck surgery: official journal of American Academy of Otolaryngology-Head and Neck Surgery.

[81] Selimoglu E (2007). Aminoglycoside-induced ototoxicity. Curr Pharm Des.

[82] Guthrie OW (2008). Aminoglycoside induced ototoxicity. Toxicology.

[83] Wang X, Zhou Y, Wang D, Wang Y, Zhou Z, Ma X (2023). Cisplatin-induced ototoxicity: From signaling network to therapeutic targets. Biomedicine & pharmacotherapy = Biomedecine & pharmacotherapie.

[84] Li M, Liu J, Liu D, Duan X, Zhang Q, Wang D (2021). Naringin attenuates cisplatin- and aminoglycoside-induced hair cell injury in the zebrafish lateral line via multiple pathways. J Cell Mol Med.

[85] Sullivan MJ, Rarey KE, Conolly RB (1988). Ototoxicity of toluene in rats. Neurotoxicol Teratol.

[86] Rebert CS, Sorenson SS, Howd RA, Pryor GT (1983). Toluene-induced hearing loss in rats evidenced by the brainstem auditory-evoked response. Neurobehavioral toxicology and teratology.

[87] Farulla A, Monaco E, Corrao CR, Cardoni F, Simonazzi S, Dallapiccola B (1986). Fanconi's anemia: in vitro tests for the individualization of heterozygotes. Giornale italiano di medicina del lavoro.

[88] Castellanos M, Fuente A (2016). The Adverse Effects of Heavy Metals with and without Noise Exposure on the Human Peripheral and Central Auditory System: A Literature Review. International journal of environmental research and public health.

[89] Yin JZ, E M, Chao H (2021). Population-based study of environmental lead exposure and hearing loss: a systematic review and meta-analysis. Public Health.

[90] Zhang B, Hu Y, Du H, Han S, Ren L, Cheng H (2024). Tissue engineering strategies for spiral ganglion neuron protection and regeneration. J Nanobiotechnology.

[91] Cohen BE, Durstenfeld A, Roehm PC (2014). Viral causes of hearing loss: a review for hearing health professionals. Trends Hear.

[92] Shi X, Qiu S, Zhuang W, Yuan N, Wang C, Zhang S (2017). NLRP3-inflammasomes are triggered by age-related hearing loss in the inner ear of mice. Am J Transl Res.

[93] Bradford RD, Yoo Y, Golemac M, Pugel EP, Jonjic S, Britt WJ (2015). Murine CMV-induced hearing loss is associated with inner ear inflammation and loss of spiral ganglia neurons. PLoS Pathog.

[94] Li X, Shi X, Wang C, Niu H, Zeng L, Qiao Y (2016). Cochlear Spiral Ganglion Neuron Apoptosis in Neonatal Mice with Murine Cytomegalovirus-Induced Sensorineural Hearing Loss. J Am Acad Audiol.

[95] Okalany NRA, Mukunya D, Olupot-Olupot P, Chebet M, Okello F, Weeks AD (2025). Postnatal cytomegalovirus infection and its effect on hearing and neurodevelopmental outcomes among infants aged 3-10 months: A cohort study in Eastern Uganda. PLoS One.

[96] Chatzakis C, Sotiriadis A, Dinas K, Ville Y (2023). Neonatal and long-term outcomes of infants with congenital cytomegalovirus infection and negative amniocentesis: systematic review and meta-analysis. Ultrasound in obstetrics & gynecology: the official journal of the International Society of Ultrasound in Obstetrics and Gynecology.

[97] Severini C (2022). Neurotrophic Factors in Health and Disease. Cells.

[98] Pettingill LN, Richardson RT, Wise AK, O'Leary SJ, Shepherd RK (2007). Neurotrophic factors and neural prostheses: potential clinical applications based upon findings in the auditory system. IEEE transactions on bio-medical engineering.

[99] Gao WQ (1998). Therapeutic potential of neurotrophins for treatment of hearing loss. Mol Neurobiol.

[100] Fariñas I, Jones KR, Backus C, Wang XY, Reichardt LF (1994). Severe sensory and sympathetic deficits in mice lacking neurotrophin-3. Nature.

[101] Staecker H, Kopke R, Malgrange B, Lefebvre P, Van de Water TR (1996). NT-3 and/or BDNF therapy prevents loss of auditory neurons following loss of hair cells. Neuroreport.

[102] Yu Q, Liu S, Guo R, Chen K, Li Y, Jiang D (2024). Complete Restoration of Hearing Loss and Cochlear Synaptopathy via Minimally Invasive, Single-Dose, and Controllable Middle Ear Delivery of Brain-Derived Neurotrophic Factor-Poly(dl-lactic acid-co-glycolic acid)-Loaded Hydrogel. ACS Nano.

[103] Leake PA, Akil O, Lang H (2020). Neurotrophin gene therapy to promote survival of spiral ganglion neurons after deafness. Hear Res.

[104] Qi J, Tan F, Zhang L, Lu L, Zhang S, Zhai Y (2024). AAV-Mediated Gene Therapy Restores Hearing in Patients with DFNB9 Deafness. Advanced science (Weinheim, Baden-Wurttemberg, Germany).

[105] Kux M, Coalson JJ, Massion WH, Guenter CA (1972). Pulmonary effects of E. coli endotoxin: role of leukocytes and platelets. Ann Surg.

[106] Dufner-Almeida LG, Cruz DBD, Mingroni Netto RC, Batissoco AC, Oiticica J, Salazar-Silva R (2019). Stem-cell therapy for hearing loss: are we there yet?. Braz J Otorhinolaryngol.

[107] Hu Z, Ulfendahl M (2013). The potential of stem cells for the restoration of auditory function in humans. Regen Med.

[108] Clarke DL, Johansson CB, Wilbertz J, Veress B, Nilsson E, Karlström H (2000). Generalized potential of adult neural stem cells. Science (New York, N.Y.).

[109] Dabdoub A, Nishimura K (2017). Cochlear Implants Meet Regenerative Biology: State of the Science and Future Research Directions. Otology & neurotology: official publication of the American Otological Society, American Neurotology Society [and] European Academy of Otology and Neurotology.

[110] Yokota S, Yoshimura H, Shirai K, Kanaya K, Adachi Y, Fujinaga Y (2023). Feasibility and limitations of head MRI in patients with cochlear implants. Auris, nasus, larynx.

[111] Jeschke M, Moser T (2015). Considering optogenetic stimulation for cochlear implants. Hear Res.

[112] Jinka S, Wase S, Jeyakumar A (2023). Complications of cochlear implants: a MAUDE database study. The Journal of laryngology and otology.

[113] Wilson BS (1997). The future of cochlear implants. British journal of audiology.

[114] Schwartz MS, Wilkinson EP (2017). Auditory brainstem implant program development. The Laryngoscope.

[115] Dieter A, Keppeler D, Moser T (2020). Towards the optical cochlear implant: optogenetic approaches for hearing restoration. EMBO Mol Med.

[116] Wei J, Wang W, Zhai R, Zhang Y, Yang S, Rizak J (2016). Neurons Differentiated from Transplanted Stem Cells Respond Functionally to Acoustic Stimuli in the Awake Monkey Brain. Cell Rep.

